# Supplementary material for: Entrepreneurial ecosystems in cities: The role of institutions
Source: PLoS One. 2021 Mar 8;16(3):e0247609. doi: 10.1371/journal.pone.0247609 (PMC7939368; doi:10.1371/journal.pone.0247609)
Supplement: S1 File — (DOCX) [file pone.0247609.s001.docx]

Data Availability Statement

Entrepreneurship ecosystem data includes 1652 observations across 16 cities and nine countries (transition economies). Variables, descriptions, data sources, and descriptive statistics for the study variables are summarized in Table 1 . data was collected via online survey .

Our other sources were the Times Higher Education of the Global University Ranking to identify the number of business schools and management departments in each city as well as environmental air quality data from the IQAir Earth data (2020) for environmental awareness and industrial agglomeration in cities.

The distribution of observations is consistent across cities in our sample: Kyiv, Ukraine (7.26%), Lviv, Ukraine (5.81), Wroclaw, Poland (6.17%), Warsaw, Poland (6.17 %), Batumi, Georgia (3.63%), Tbilisi, Georgia (7.81%), Astana (6.17%) and Almaty in Kazakhstan (6.36%), Cluj, Romania (6.96%), Bucharest, Romania (7.20%), Istanbul, Turkey (5.45%), Sarajevo (Bosnia and Herzegovina (6.23%), Zagreb (6.96%) and Osijek, Croatia (6.3%), Sofia (5.93%) and Plovdiv, Bulgaria (5.51%). Almost 95% of respondents have a university degree and above.

Our four major groups of stakeholders are entrepreneurs (35.1%), university professors (8.1% of a sample), policymakers (7.4% of a sample), as well as respondents of multiple affiliations (31.9% of a sample). Other stakeholders include investors, a representative from the chamber of commerce, managers in multinational firms, technology transfer office (TTO) managers, managers in techno park, journalists, managers in business incubators, and lawyers.

Datafile in stata format and dofiles are provided for replication and attached to the submission
