# Supplementary material for: Entrepreneurial ecosystems in cities: The role of institutions
Source: PLoS One. 2021 Mar 8;16(3):e0247609. doi: 10.1371/journal.pone.0247609 (PMC7939368; doi:10.1371/journal.pone.0247609)
Supplement: Attachment — Submitted filename: Review of Polis V2.docx [file pone.0247609.s005.docx]

Review of the paper „Entrepreneurial Ecosystems in Cities: The Role of Institutions”

The paper has improved a lot as compared to the previous version. I am satisfied with the changes and have no further suggestions.
